# A content and quality analysis on open online platform videos on do not resuscitate (DNR)

**DOI:** 10.1038/s41598-026-46624-5

**Published:** 2026-04-15

**Authors:** Elina Ahonen, Reino Pöyhiä, Minna Hökkä, Maria Valjus, Minna Peake, Eeva Pyörälä, Timo Carpén

**Affiliations:** 1 South Savo Wellbeing Services County, Mikkeli, Finland; 2 Department of Clinical Medicine, University of Eastern Finland, Kuopio, Finland; 3 Diaconia University of Applied Sciences, Oulu, Finland; 4 Palliative Care Center, Comprehensive Center, Helsinki University Hospital and University of Helsinki, Helsinki, Finland; 5 Wellbeing Services County of North Karelia, Joensuu, Finland; 6 Centre for University Teaching and Learning, Faculty of Educational Sciences, University of Helsinki, Helsinki, Finland

**Keywords:** Do not resuscitate, DNR, Quality, Video, Content, Decision-making, Health care, Medical research

## Abstract

The decision to implement a Do Not Resuscitate (DNR) order is a challenging medical decision made by physicians. Both laypersons and healthcare professionals need a better understanding of DNR orders. Educational videos could enhance DNR understanding, but their content and quality haven’t been systematically evaluated. A search on YouTube and Google identified 90 eligible videos for analysis. Technical information was collected, and the content was examined using a DNR questionnaire (DNR-q) developed for this study. Video quality was evaluated with the modified DISCERN (m-DISCERN), Global Quality Scale (GQS) and JAMA scale. Most videos were from the United States (n = 51, 56.7%) and presented by a self-described physician (n = 45, 50%). The median DNR-q and m-DISCERN scores were 10/23 and 38/80, respectively. Higher scores on DNR questionnaire correlated significantly (*p* < 0.001) with higher scores on m-DISCERN, GQS laypersons and GQS professional scores. However, only 20 (22%) videos were ranked good or excellent with m-DISCERN, and none covered all DNR-q items. Only 12 (13.3%) videos achieved the maximum JAMA score of 4. The content and quality of the videos were suboptimal. Most videos failed to provide acceptable information regarding DNR decision-making. Better planning and evidence-based approaches are needed to improve the quality of DNR-related videos published on open online platforms.

**Supplementary Information:**

The online version contains supplementary material available at 10.1038/s41598-026-46624-5.

## Introduction

Digital and open online educational resources have become an important source of information for healthcare professionals and laypersons, complementing formal education and clinical guidelines. Video-based content is increasingly used to support learning related to clinical procedures, communication skills, and complex ethical decision-making^1^. Publicly available platforms provide easy access to educational videos on cardiopulmonary resuscitation (CPR), advance care planning (ACP), and Do-Not-Resuscitate (DNR) decisions; however, the quality and reliability of such content are not routinely verified.

Survival after cardiopulmonary resuscitation (CPR) has not significantly improved over the past 10 years^2^. Out-of-hospital cardiac arrest 30-day survival rate after CPR is, on average, 11.7% in Europe^3^. In addition, the functional capacity and quality of life of the survived patients fail to return to pre-arrest levels^4–6^. Previous studies have shown a negative association between poor outcome after CPR and increased numbers of comorbidities^7–9^. These findings suggest that withholding CPR should occur more frequently, highlighting the necessity of making Do-Not-Resuscitate decision (DNR) decisions whenever appropriate.

According to a recent scoping review, barriers to effective DNR decisions include ethical uncertainty in the decision-making process, poor communication between patients, families and healthcare providers, and unclear or lacking DNR instructions^10,11^. In a questionnaire survey, Gibbs et al.^10^ reported that only 53% of the 43 surveyed countries across all continents had national guidelines for making DNR orders. Another survey examining 48 National Health Service trusts in the UK found substantial inconsistency in the DNR guidelines^12^. A recent Swedish analysis of 3,583 DNR patient forms showed that in 90% of cases, physicians did not comply with national legislation regarding DNR orders^13^.

Despite significant international variation in the DNR orders, several core principles are consistently emphasised across national and international guidelines. These principles directly address common misconceptions surrounding DNR decision-making, particularly the erroneous belief that a DNR order implies limitation of all medical treatment (as summarised in Table 1), current guidelines explicitly state that (1) a DNR order must be documented in the patient’s medical records and regularly reviewed, (2) a DNR order applies solely to cardiopulmonary resuscitation and does not preclude other appropriate medical treatments and (3) DNR-decision making should be integrated into advanced care planning (ACP), as recommended by the European Resuscitation Council^14^ and the American Heart Association (AHA) guideline^15^. Alarmingly, according to the recent studies, even 30–70% of healthcare professionals misunderstand these basic concepts and wrongly consider DNR meaning such as withholding parenteral medications or limiting broad use of other resources^16,17^.

**Table 1:** DNR decision according to national guidelines.

	Finland (Valvira)	UK (NHS)	India	US (AMA)	HONG KONG
Meaning	Do not attempt cardiopulmonary resuscitation	Do not attempt cardiopulmonary resuscitation	Do not attempt cardiopulmonary resuscitation	Do not attempt cardiopulmonary resuscitation	Do not attempt cardiopulmonary resuscitation
DNR in relation to other care	Other care and treatment are still given	Other care and treatment are still given	Other care and treatment are still given	Other care and treatment are still given	Other care and treatment are still given
Decision making	Medical decision made by a physician	Medical decision made by a physician	Medical decision made by a physician	Patient’s decision, DNR order: physician	Medical decision made by a physician
Discussion and information with patient and closest ones	Should be discussed with the patient and her/his closest ones. The will of the patient should be clarified	Form filled, consent not needed. Patient and closest ones should be informed and the decision explained	Should be discussed and explained to the patient and the closest ones	DNR discussion early, while the patient has decision-making capacity. Discussion and information with the patient and closest ones	Discussion and information with the patient and the closest ones
Ethics	No	No	Yes	Yes	Yes
ACP (advanced care planning)	Recommended	Recommended	Recommended	No	Recommended
Target patients	Patients with incurable disease/condition/EOLcare	Patients with incurable disease/condition + frail, approaching end of life	Patients with incurable disease/condition/ EOLcare, survival is not expected	Any patient medically at risk of cardiopulmonary arrest, regardless of the patient’s age or whether the patient is in EOL care within the constraints of applicable law	Patients with incurable disease/condition/EOL care
Documentation	Medical records: individuals present, medical reasoning	Special form, medical records	Special form, medical records	Medical records	Medical records
Duration of dnr decision	Continuous or temporary Decision must be reviewed in a new care unit or if the condition improves	Continuous Decision must be reviewed in a new care unit	Continuous	Revised if circumstances change (such as clinical state, care unit, patient’s wishes)	Revision at least once every 12 months or shorter revision period if clinically appropriate Invalid without revision!
Cancellation policy	Yes	Yes	Yes	Yes	Yes
Information about cpr	N/A	Procedures for restarting the heart after it stops, successful in 1–2/10	Emergency procedures for restarting the heart after it stops risks: neurological injury, dependency on others, vegetative state, rib fracture	Emergency standard procedures for restarting the heart after it stops, success depends on survival for 24 h	Invasive medical therapy to support ventilation and circulation when cardiac arrest occurs risk of neurological impairment for survivors
Examples when DNR decision is made	End of life care and part of ACP	N/A	N/A	N/A	Adults with: terminal illness, irreversible coma or persistent vegetative state, other end-stage irreversible life limiting condition
References	Valvira 2020^18^	Perkins et al. 2016^19^	ICMR 2020^4^	AMA 2024^20^	HA 2020^21^

Valvira = National Supervisory Authority for Welfare and Health, NHS = National Health Service, AMA = American Medical Association, ACP = advance care plan, EOL = end-of-life.

There is a paucity of studies on how medical students and physicians are educated about DNR decision-making and how patients are adequately informed about this process. Public media is a significant source of medical information for laypersons, including information about CPR^22–24^. Additionally, healthcare professionals also use open online resources as a source of professional information^22,24^. However, a major concern is the reliability of both public and traditional media content. For example, in television series survival rates after resuscitation are often portrayed as higher than they are in reality^25^. Previous studies have shown that many people have unrealistic expectations about the CPR procedure and the likelihood of survival^26,27^. On the other hand, evidence suggests that after a thorough discussion with healthcare professionals or watching a well-produced educational video on CPR/DNR, many individuals reconsider their preferences and choose to have a DNR order^28–30^.

Both layperson and healthcare professionals need to improve their knowledge and understanding of the advantages and disadvantages of CPR and DNR to make timely and well-informed decisions based on comprehensive discussions^27,30^. We hypothesise that currently available educational videos on DNR decision-making could help healthcare professionals initiate the conversation more timely. However, the quality of DNR-related content on publicly available online videos has not been systematically studied. The aim of this research is to evaluate the quality of educational DNR videos available on the internet.

## Methods

### Screening of the videos

In this study, we analysed videos published on the open online video-sharing platforms. The video search was performed independently by two authors (EA, TC) on the same days in June 2023. The online search was conducted using the widely used open-access search platforms YouTube and Google, with the following keywords: “DNR”, “DNAR”, “DNACPR”, “do not resuscitate”, “do not attempt resuscitation”, and “do not attempt cardiopulmonary resuscitation”. Terms such as EOL (end-of-life) and ACP were excluded from the search keywords, as the focus was specifically around understanding of do-not-resuscitate. A total of 270 videos matching the criteria were identified. Only videos in English language were included. Exclusion criteria included commercial and duplicate videos, videos focusing on patient’s/caregiver’s personal experiences, videos that were not exclusively about DNR/DNAR and videos specifically about DNR for paediatric patients. The final analysis was performed on 90 videos (Supplementary Table S1).

### Structure of the videos

Technical data were collected for all videos, including the internet address, date of upload, duration, number of likes, views, the person(s) featured in the video, as well as presence of graphic content and on-screen text. The popularity of the videos was calculated with Video power index (VPI) = like ratio * view rate /100^32^. Four experienced physicians and two nurses (EA, MV, TC, RP, MH, MP) well acquainted with clinical, ethical and legal aspects of DNR decisions independently evaluated the videos using the following predefined tools: modified DISCERN, DNR content questionnaire (DNR-q), Global Quality Scale (GQS) and JAMA criteria. A detailed description of these tools is provided below.

### Content analysis

We developed an assessment tool (DNR-q) to evaluate the content of the videos based on national guidelines of DNR decision-making, as outlined in Table 1. The DNR-q (Table 2) included 23 items, pre-selected by the authors (EA, RP, TC). The tool used dichotomous scoring (yes = 1, no = 0), with a maximum score of 23.

**Table 2:** The DNR questionnaire.

Item	Content (1 = yes, 0 = no)
1. What DNR stands for	Do not attempt cardiopulmonary resuscitation
2. Excluding treatment other than CPR	DNR does not rule out other treatments modalities than CPR
3. Who makes the DNR decision (part 1)	Physician
4. Who makes the DNR decision (part 2)	If mentioned that (1) the patient or (2) the physician and patient together make the decision
5. When is a DNR decision made?	Terminally or seriously ill patient for whom resuscitation would do more harm than good (USA: always when the patient wants it)
6. Palliative care/ Advance Care Planning	If advance care planning is mentioned
7. How the decision is made	Physician discusses the decision with the patient
8. Ethics: patient’s will	DNR is a physician’s decision, yet the patient’s will should be heard
9. Ethics: patient’s will in a situation when the patient cannot communicate	How the patient’s will is clarified if he/she cannot communicate. Discussion with the closest ones/trustee about what the patient’s will would be
10. Documentation in the patient’s medical records	DNR decision must be written in the patient’s medical records
11. Patient signs the decision/form	If mentioned, that it is recommended that the patient signs the decision
12. Validity of the decision	The validity of the DNR decision must be reviewed regularly
13. Review of the validity of the decision	The validity of the decision must be reviewed at regular intervals
14. The transfer of the DNR decisionin documents with the patient	The documentation of the DNR decision must follow if the patients moves or the care unit changes
15. The reversal of the DNR decision	The reversal of the DNR decision when the patient’s condition significantly improves
16. What does CPR means	Cardiopulmonary resuscitation and/or defibrillation
17. When CPR is started	If the patient stops breathing/breathing is not normal or the heart stops beating, when lack of signs of life is diagnosed
18. Aims of CPR	To restart the heart beating
19. Advantages vs. disadvantages of CPR	May restart heart beating/breathing Short-term and long-term complications, e.g. neurological symptoms and rib fractures may occur
20. Legal aspect	If mentioned any of the following: (a) the patient can consult a lawyer (e.g. to get more information), (b) DNR decision does not conflict with laws (c) withholding CPR is not illegal if the patient wishes so
21. Interaction	If mentioned interaction and discussion with the patient and closest ones in any form
22. Ethical issues	The physician and the patient (or closest one) may have a different opinion/understanding
23. Where to get further information	If mentioned how to get further information in any way

One point, if an item is addressed in the video, 0 point, if it is not addressed in the video.

Abbreviations: p = point, CPR = cardiopulmonary resuscitation, DNR = do not resuscitate

Content validity was assessed independently by five healthcare professionals with substantial experience in resuscitation and DNR decision-making in Finland. These professionals were not involved as authors in the present study. Each expert ranked both the relevance and clarity of the items on a 1–4 scale, where 4 indicated a clear or highly relevant item. The Content Validity Index (CVI) for relevance (CVIR) and clarity (CVIC) was calculated as follows: CVI = [number of experts ranking 3–4] / [total number of recruited experts]. Items were revised based on the reviewers’ feedback. The average values of CVIR and CVIC for the whole questionnaire were 0.96 and 0.90, respectively demonstrating acceptable relevance and clarity^32^.

### Quality of the videos

*Modified DISCERN.* DISCERN instrument has been employed to evaluate consumer health information presented in written form as well as in video format^24,33,34^. DISCERN includes 16 questions and each question is ranked with Likert scale 1 (no)—5 (yes) and the sum of the questions scores is calculated. A total DISCERN score ranks from 16 to 80. Scores 64–80 indicate excellent quality, 52–63 good quality, 41–51 average quality, 30–40 poor quality, 16–29 very poor quality^35^. For this study the content of DISCERN was adjusted to fit DNR decision yet so the purpose of the instrument remained. The content validity was assessed as explained previously. The average values for all the items in the final modified DISCERN were 1 and 0.98 for CVI_R_ and CVI_C_ respectively demonstrating acceptable relevance and clarity^32^.

*Global quality scale (GQS).* The quality of the videos was assessed using Global Quality Scale (GQS), a five-point Likert scale for evaluating item characteristics (Table 3). This tool was used to assess the suitability of the videos for both laypersons and healthcare professionals. Scores 4–5 indicate good quality, and scores 1–3 indicate poor to moderate quality.

**Table 3:** Global quality scale (GQS).

Item characteristics	Points
Poor quality; poor flow of the video; most information missing; not at all useful to patients/healthcare professionals	1
Generally poor quality and poor flow; some information listed, but many important topics missing; of very limited use for patients/healthcare professionals	2
Moderate quality; suboptimal flow; some important information adequately discussed, but other information poorly discussed; somewhat useful for patients/healthcare professionals	3
Good quality and generally good flow; most of the relevant information listed, but some topics not covered; useful for patients/healthcare professionals	4
Excellent quality and flow; very useful for patients/healthcare professionals	5

*JAMA.* Jama scoring system is used to evaluate the reliability and accuracy of video or written content. It assesses four key criteria: (1) Authorship: authors and contributors, their affiliations and relevant credentials provided; (2) Attribution: references and sources for all content clearly listed clearly, including relevant copyright information; (3) Disclosure: website “ownership”, sponsorship, advertising, underwriting, commercial funding should be fully disclosed; and (4) Currency: The date when content was posted and last updated should be indicated. Each criterion is ranked with 1 point (yes) or 0 points (no)^36^.

### Statistical analysis

Statistical analysis was performed using SPSS 25 (IBM SPSS Statistics 25, IBM, Somers, IL, USA). The Mann–Whitney U test was used to compare medians and other non-parametric data. Differences in categorical data between groups were tested using Pairwise Pearson’s chi-squared test. A p-value < 0.05 was considered statistically significant. Data are presented as absolute values, medians with percentages, and interquartile ranges (IQR) where appropriate.

## Results

### Descriptives

A total of 90 videos were included in the final analysis. The most common presenters identified themselves as physicians, and the majority of the videos were American and contained only graphical material (Table 4). Forty (44%) videos were produced by an unspecified organisation (excluding hospitals or universities), 40 (44%) by independent sources/presenters, 5 (6%) by universities and 5 (6%) by hospitals. Median time since upload was 911.5 days (IQR 2317.3) and median runtime 5.3 min, (IQR 421.95). Median number of views was 504 (IQR 3420) and median number of likes 5.5, (IQR 31).

**Table 4:** Descriptive data of the videos.

**Total number of videos (%)**	n (%)
	90 (100)
Video presenter	
Physician*	45 (50)
Unspecified	33 (36.6)
Nurse	7 (7.8)
Advocate	5 (5.6)
Country	
USA	51 (56.7)
UK	32 (35.6)
India	3 (3.3)
Other	4 (4.4)
Technical features	
Graphics and text in video	34 (37.8)
Only text in thevideo	13 (14.4)
Only graphics in the video	43 (47.8)

*refers to an MD/ an actor presenting physician.

### Content analysis

Table 5 presents the distribution of content items according to the DNR questionnaire across all videos. Topics such as validation of decision-making, continuity of DNR decision, as well as ethical and legal issues were particularly underrepresented.

**Table 5:** The distribution of content items according to the DNR questionnaire in all videos. N (%) denotes for the number (percentage of all) of videos including the clarification of the item.

Item	N (%)
1. What DNR stands for	80 (89)
2. Ruling out treatment other than resuscitation	60 (67)
3. Who makes the DNR decision (part 1)	44 (49)
4. Who makes the DNR decision (part 2)	65 (72)
5. When is a DNR decision made?	62 (69)
6. Palliative care/ advance care plannning	25 (28)
7. How the decision is made	56 (62)
8. Ethics: patient’s will	56 (62)
9. Ethics: patient’s will in a situation when the patient cannot communicate	31 (34)
10. Documentation in the patient’s medical records	46 (51)
11. Patient should sign the decision/form	29 (33)
12. Validity of the decision	8 (8)
13. Review of the validity of the decision	6 (6)
14. The transfer of the DNR decision in documents with the patient	14 (15)
15. The reversal of the DNR decision	13 (14)
16. What does CPR mean	64 (71)
17. When CPR is initiated	64 (71)
18. Aims of CPR	56 (62)
19. Advantages vs. disadvantages of CPR	37 (41)
20. Legal aspects	23 (26)
21. Interaction	65 (71)
22. Ethical issues	28 (31)
23. Where to get further information	19 (21)

The median DNR-q score was 10.0 (IQR 21) out of a maximum possible score of 23, indicating that, on average, fewer than half of the predefined content items were addressed per video. A total of 13 videos (14%) achieved score of 15 or higher (65–100%) which was considered to indicate reasonably acceptable content coverage. None of the videos attained the maximum DNR-q score 23 suggesting that no single video provided comprehensive coverage of all key aspects of DNR decision-making.

### Quality

The median modified DISCERN (m-DISCERN) score of all videos was 38 (IQR 20.4) out of a maximum of 80. According to established cut-off values, this corresponds to poor overall quality. Only 20 videos (22%) scored 52 or higher, indicating good quality (65–100%). None of the videos achieved maximum scores (80) on m-DISCERN.

The median GQS score was 2.5 (IQR 1) for both laypersons and healthcare professionals. Given that the GQS ranges from 1 (poor quality) to 5 (excellent quality), these median values indicate low to moderate perceived usefulness of the videos for both target audiences.

Regarding the JAMA criteria, authorship was identified in 66 (73%) videos, attribution in 14 (16%), disclosure in 44 (49%) and currency in 80 (89%) videos. Only 12 (13.3%) videos achieved the highest possible JAMA score of 4 indicating that the majority of videos lacked key elements required for transparency and reliability. Eight (8.9%) videos scored 0 points. The median VPI was 21.28 (IQR 1015.2) reflecting wide variability in viewer engagement across videos.

### Correlations

The median run time of the videos was longer in the good-score group of m-DISCERN compared to the poor-score group. Higher median scores on DNR-q correlated significantly (*p* < 0.001) with higher m-DISCERN scores as well as higher GQS scores for both laypersons and professionals (Table 6) indicating that videos with more comprehensive content also tended to be of higher overall quality. However, good scores on m-DISCERN and DNR-q were not associated with the presenter. A greater proportion of British videos were in the good-score groups of the m-DISCERN and DNR-q and they had higher median scores compared to American videos.

**Table 6:** Correlations between demographic factors and quality and content tools. Median values (with IQ deviations) or percentages are given.

	m-DISCERN good–excellent (n = 20)	m-DISCERN poor-very poor (n = 70)	p-value	Missing (%)	Median DNR-q points (IQR)	p-value
Median run time in minutes (IQR)	646 (560)	210 (259)	** < 0.001**	8 (8.9)	N/A	N/A
Country, n (%)				1 (1.1)		
USA^a^	7 (13.5)	45 (86.5)	**0.018**^ab^		9.3 (6)	**0.005**^ab^
UK^b^	12 (36.4)	21 (63.6)	0.470^ac^		13.5 (7.3)	0.470^ac^
Other^c^	1 (25.0)	3 (75.0)	1.000^bc^		12.3 (11.9)	0.759^bc^
Presenter, n (%)			0.367	0		0.625
Physician	12 (26.1)	34 (73.9)			10 (8.3)	
Other	8 (18.2)	36 (81.8)			9.5 (6.8)	
Median DNR-q points (IQR)	14 (2.9)	9 (6.4)	** < 0.001**	0	N/A	N/A
GQS laypersons, n (%)			** < 0.001**	0		** < 0.001**
High points, ≥ 3	20 (45.5)	24 (54.5)			13.5 (2.9)	
Low points, < 3	0 (0)	46 (100)			6.5 (4.8)	
GQS professionals, n (%)			** < 0.001**	0		** < 0.001**
High points, ≥ 3	20 (47.6)	22 (52.4)			14 (2.8)	
Low points, < 3	0 (0)	48 (100)			6.8 (4.9)	

m-DISCERN = modified DISCERN, DNR-q = do not resuscitate questionnaire, GQS = global quality scale, IQR = interquartile range. An asterix^abc^ indicates pairwise comparisons. *P*-values < 0.05 are shown in bold.

## Discussion

The main findings of this study on YouTube and Google videos related to DNR decision-making indicate that the majority of these videos feature inadequate content for both laypeople and healthcare professionals. In addition, the overall quality of the videos was poor. Most videos were produced in the USA and presented by individual physicians. One third of the videos were European, all originating from the United Kingdom. Only a few videos were produced by universities or hospitals. To our knowledge, this is the first study to evaluate the quality and content of open-access online video resources related to DNR decision-making.

Our findings align with the previous studies about CPR videos in online video resources, which have also been criticised for inadequate content and poor quality and not meeting the guidelines’ criteria^37–39^. Similar observations have been made regarding publicly available online videos on palliative care and advance care planning^38^ and other medical issues^24,34,40–45^.

Our findings are concerning but not surprising, given that only 11% of the videos evaluated in the current study were produced by hospitals or universities. Additionally, in our study, the video presenter did not correlate with scores on DISCERN or DNR-q. The systematic review process for open online videos appears to be unknown or absent, which may explain our results. Previous studies have shown that videos produced by healthcare organisations had better quality than those produced by non-healthcare organisations, however, quality still varies, and the overall quality remains poor^34,43^. The higher content and quality scores of UK videos compared with US videos may reflect differences in national legal and regulatory frameworks, with more standardised DNACPR policies in the UK and greater variability in the US.

In our study, longer video duration correlated significantly with better quality compared to shorter videos. Li et al.^41^ found a similar correlation between video duration and quality. Yet, longer duration does not guarantee the usefulness of the video. Research has shown that educational videos shorter than 6 min achieve nearly 100% engagement among medical students, whereas videos lasting 12–40 min see a significant drop in engagement, down to approximately 20%^46^.

Videos publicly available on online platforms have an important impact on the accumulation of knowledge for both laypersons and healthcare professionals. However, previous studies have shown that YouTube is not a reliable source of medical and health-related information^22,43^. On the other hand, studies have also shown that carefully made CPR or ACP-related videos can enhance viewers’ knowledge and facilitate decision-making^29,46–48^. While the internet and YouTube are valuable sources of information, it should be kept in mind that the reliability of the content is not necessarily verified. Perhaps, further studies could focus on improving the quality of the videos produced by a university, medical society or a dedicated group of specialists. In addition, more regulation could be considered on the responsibilities of open online platform organisations to improve pre-validation and quality of the published videos.

This study offers several notable strengths. It is the first to evaluate videos specifically focused on DNR decision-making, addressing a significant gap in the literature. The other merit is the development of analysing tools. In the absence of appropriate measures and criteria for assessment of online videos^40^, we developed new instruments for analysis. First, we created the “DNR-q” tool for evaluating the content of the videos and examined its content validity. We collected information about meaning and decision-making process in DNR order in various countries^4,18–21^ (Table 1) and used it for defining the optimal content of a DNR-decision. Our instrument appeared to have a good content validity, and we consider it feasible in assessing process. When comparing international recommendations, the American DNR guidelines seem to emphasise more the role of an individual patient in decision-making than guidelines from other countries possibly due to a different legal system.

Secondly, we used previously validated scales DISCERN, JAMA and GQS to assess the quality of the videos and adjusted them appropriately for our analysis^43^. These tools are widely recognised for evaluating the quality and reliability of health-related information in videos. In our study, higher median scores on DNR-q correlated significantly with higher scores on Discern, GQS laypersons and GQS professional indicating, too, that DNR-q is a usable tool for evaluating DNR video content.

However, there are limitations to consider. We used medical terminology in the search terms, which may not correspond to the terms commonly used by laypersons. All individuals who evaluated the videos were Finnish healthcare professionals (physicians or nurses), with expertise in DNR decision-making, which may have influenced their interpretation of the videos’ quality, particularly across different national contexts. We did not conduct a systematic search on DNR guidelines but collected information available on national guidelines from different countries across the world. In addition, the systematic evaluation of the validity and reliability of DNR-q remains to be done. The study was limited to English-language videos and focused solely on YouTube videos and open-access videos through Google search, potentially limiting the diversity of video content analysed. Furthermore, a recent systematic review highlighted the absence of standardised measures and grading criteria for evaluating social media videos, which may impact the consistency and comparability of our findings^43^.

## Conclusions

This study reveals that most English-language YouTube videos on DNR orders contain insufficient content and are of poor to moderate quality. Notably, no significant relationship was observed between a video’s length and its’ content quality. Given the growing reliance on open online platforms as a source of medical information, there is a clear need for standardised frameworks, expert review processes, and validated quality criteria to guide video development prior to publication. We advocate for medical institutions, particularly medical schools, to spearhead this initiative, as publicly available online videos remain a pivotal resource for both healthcare professionals and laypersons.

## Supplementary Information

Supplementary Information.

## Data Availability

The datasets generated during and/or analysed during the current study are available from the corresponding author on reasonable request.
